# Risk of macrovascular events among patients with ICD-defined neuromyelitis optica in Taiwan

**DOI:** 10.3389/fneur.2026.1799026

**Published:** 2026-07-01

**Authors:** Chih-Jaan Tai, Kuang-Hua Huang, Tsuei-Hung Wang, Tung-Han Tsai, Shuo-Yan Gau, Vincent Ping-Sheng Lai, Zheng-Ren Lin, Chien-Ying Lee

**Affiliations:** 1 Department of Otorhinolaryngology-Head and Neck Surgery, China Medical University Hospital, Taichung, Taiwan; 2 Department of Health Services Administration, China Medical University, Taichung, Taiwan; 3 Department of Healthcare Administration, Asia University, Taichung, Taiwan; 4 Department of Medical Education, Ditmanson Medical Foundation Chia-Yi Christian Hospital, Chiayi, Taiwan; 5 School of Medicine, Chung Shan Medical University, Taichung, Taiwan; 6 Department of Pharmacology, Chung Shan Medical University, Taichung, Taiwan; 7 Department of Pharmacy, Chung Shan Medical University Hospital, Taichung, Taiwan

**Keywords:** cardiovascular disease, cerebrovascular disease, macrovascular events, neuromyelitis optica, real-world data

## Abstract

**Background:**

An elevated risk of cardiovascular disease (CVD) and cerebrovascular disease (CBD) has been observed in patients with ICD-defined Neuromyelitis Optica (hereinafter referred to as NMO). However, population-based research on this topic remains limited. In this study, the risk of macrovascular events was compared between individuals with NMO and a matched population without NMO in Taiwan.

**Methods:**

Data for this retrospective cohort study were collected from a nationwide database in Taiwan. A total of 1,376 patients with new-onset NMO between 2003 to 2020 were enrolled. A Cox proportional hazards model was constructed to investigate CVD and CBD risk in patients with NMO and controlled for relevant variables.

**Results:**

After relevant variables were controlled for, patients with NMO exhibited a significantly higher risk of CVD (adjusted hazard ratio [aHR] = 1.40; 95% confidence interval [CI] = 1.11–1.77) and CBD (aHR = 3.37; 95% CI = 2.69–4.22) than matched controls. Significant associations were observed between NMO and ischemic stroke, hemorrhagic stroke, and transient ischemic attack but not acute myocardial infarction, atrial fibrillation, coronary artery disease, or heart failure.

**Conclusion:**

Individuals with NMO exhibited an elevated risk of CBD. Conversely, NMO was not associated with an elevated risk of certain CVDs.

## 1 Introduction

Neuromyelitis optica spectrum disorder (NMOSD) is characterized by immune-mediated demyelination and axon damage, predominantly affecting the optic nerves and spinal cord (1). Clinically, NMOSD often presents with severe acute transverse myelitis, optic neuritis, and/or encephalitis involving the brain or brainstem (2, 3). In 2004, the highly specific autoantibody NMO immunoglobulin G (NMO-IgG) was discovered (4, 5). NMOSD was previously thought to be associated with both AQP4-antibodies and MOG-antibodies (6, 7). MOG antibody-associated disease (MOGAD) is now recognized as a distinct entity (8, 9). AQP4 antibodies drive astrocytic injury and blood–brain barrier (BBB) dysfunction in NMOSD, whereas MOGAD involves a separate demyelinating process targeting myelin oligodendrocyte glycoprotein (10–12). Accordingly, they exhibit distinct clinical courses, treatment requirements, and prognoses, a distinction that is, unfortunately, not captured by administrative claims data based on historical ICD coding.

Although a postmortem pathological study revealed a positive correlation between MS and the burden of cerebral small vessel disease (13), our understanding of the vascular alterations in the brain associated with NMO remains limited. A small number of cerebrovascular disease (CBD) cases have been reported in patients with NMO (14, 15). Additionally, a study conducted in Korea indicated that patients with NMO have a higher risk of CBD (16). Another Korean study revealed that the incidence of cardiovascular diseases (CVD), such as myocardial infarction, was elevated in individuals with NMO (17). The risk of macrovascular events in NMO remains incompletely understood and insufficiently studied. To address this gap, we investigated the association between NMO and the risk of macrovascular events in a large, population-based dataset from Taiwan. The data were sourced from Taiwan’s National Health Insurance Research Database (NHIRD). Due to the inherent limitations of historical ICD coding, which preclude a clear distinction between NMOSD and MOGAD, the target population of this study is operationally characterized as ICD-defined neuromyelitis optica (hereinafter referred to as NMO).

## 2 Materials and methods

### 2.1 Data sources

This study conducted a retrospective cohort analysis using the National Health Insurance Research Database (NHIRD) of Taiwan, spanning from 2002 to 2022. Administered by the Health and Welfare Data Science Center (HWDC), the NHIRD encapsulates the medical records of nearly 99% of Taiwan’s population under the mandatory National Health Insurance program. Clinical diagnoses were identified utilizing the *International Classification of Diseases, Ninth and Tenth Revisions, Clinical Modification (ICD-9-CM and ICD-10-CM)*. The dataset is a well-established resource for generating real-world evidence to guide clinical practice and healthcare policy (18, 19).

### 2.2 Ethics approval

This study conducted a secondary data analysis of information obtained from the NHIRD, which is maintained by the HWDC. To ensure patient privacy, the NHIRD provides scrambled random identification numbers for all insured individuals. All data were anonymized to protect participant confidentiality. Because the database contains only deidentified data, the requirement for informed consent was waived. The study protocol was approved by the Institutional Review Board of Chung Shan Medical University Hospital, Taiwan (No. CSMUH CS1-24227).

### 2.3 Study participants

**Figure 1:**
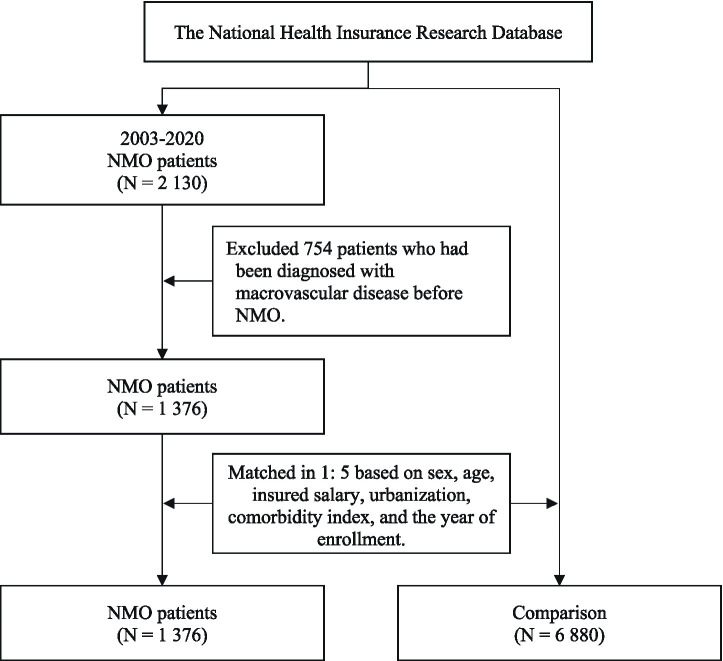
The flowchart of participant selection. NMO, ICD-defined neuromyelitis optica.

We identified patients with newly diagnosed NMO were identified based on the ICD-9-CM 341.0 and ICD-10-CM G36.0 between 2003 and 2020. NMO was defined by the presence of more than three outpatient main diagnoses or one or more inpatient primary/secondary diagnoses within one year to ensure diagnostic validity. We excluded individuals with a history of macrovascular diseases prior to their NMO diagnosis to establish a clean baseline. To mitigate potential selection bias and confounding in this observational design, a 1:5 propensity score matching (PSM) protocol was implemented. The matching criteria included sex, age, insured premium, urbanization level, Charlson Comorbidity Index (CCI), and year of enrollment. After matching, the sample included 1,376 patients with NMO and 6,880 matched general patients for comparison (Figure 1).

### 2.4 Study design

The primary objective was to evaluate the association between NMO and the subsequent risk of CVD and CBD. The CVDs investigated comprised acute myocardial infarction (*ICD-9-CM* 410; *ICD-10-CM* I21-I22), atrial fibrillation (*ICD-9-CM* 427.31; *ICD-10-CM* I48.0, II48.1, I48.2, I48.91), coronary artery disease (*ICD-9-CM* 410–414; *ICD-10-CM* I20-I25), and heart failure (*ICD-9-CM* 428; *ICD-10-CM* I50). The CBDs investigated comprised ischemic stroke (*ICD-9-CM* 433–435, 437; *ICD-10-CM* G45, G46, I63, I65-I67, I69), hemorrhagic stroke (*ICD-9-CM* 430–432; *ICD-10-CM* I60-I62), and transient ischemic attack (TIA; *ICD-9-CM* 435.9; *ICD-10-CM* G45.9). The date of diagnosis of NMO was defined as the index date for participants in the study group, and after matching, the same date was assigned as the index date for the corresponding members of the control group. All participants were tracked from the index date and continued until the occurrence of a vascular event, death, or the end date of 2022. Baseline comorbidities, such as diabetes mellitus (*ICD-9-CM* 250; *ICD-10-CM* E08-E13), hypertension (*ICD-9-CM* 401–405; *ICD-10-CM* I10-I13, I15), hyperlipidemia (*ICD-9-CM* 272; *ICD-10-CM* E78), chronic obstructive pulmonary disease (COPD; *ICD-9-CM* 490–492, 494–496; *ICD-10-CM* J40-J44), depression (*ICD-9-CM* 296.2, 296.3; *ICD-10-CM* F32, F33), anxiety (*ICD-9-CM* 300.0; *ICD-10-CM* F40-F41), sleep disturbance (*ICD-9-CM* 780; *ICD-10-CM* G47.9), and migraine (*ICD-9-CM* 346; *ICD-10-CM* G43, G44), were accounted for in the analysis.

### 2.5 Statistical analysis

All statistical procedures were performed using SAS software (version 9.4), with significance set at *p* < 0.05. Chi-square tests were utilized to compare baseline characteristics between groups. We employed Cox proportional hazards models to estimate adjusted hazard ratios (aHRs) and 95% confidence intervals (CIs) for CVD and CBD, controlling for all identified covariates. Furthermore, subgroup analyses were conducted based on NMO severity, categorized by the proportion of length of stay in the hospital and the hospitalization records.

## 3 Results

Table 1 presents the baseline characteristics of the study population. Following PSM, the NMO and control cohorts were well-balanced regarding demographic variables and CCI scores (*p* > 0.05). Notably, the NMO group exhibited a lower prevalence of diabetes and COPD but a significantly higher frequency of hyperlipidemia, depression, anxiety, insomnia, and migraines compared to the control group.

**Table 1:** Distribution of baseline characteristics after matching.

Variables	Total		Comparison		NMO ^b^		*p*-value
	*N*	%	*N*	%	*N*	%	
Total	8,256	100.00	6,880	100.00	1,376	100.00	
Sex^a^							0.756
Female	6,261	75.84	5,222	75.90	1,039	75.51	
Male	1,995	24.16	1,658	24.10	337	24.49	
Age (year)^a^							0.999
<25	1,515	18.35	1,261	18.33	254	18.46	
25–34	1,674	20.28	1,395	20.28	279	20.28	
35–44	2,177	26.37	1,814	26.37	363	26.38	
≥45	2,890	35.00	2,410	35.03	480	34.88	
Mean ± SD	41.55 ± 18.92		41.96 ± 19.58		39.52 ± 14.98		
Insured salary (NTD)^a^							0.997
≤21,000	2,082	25.22	1,735	25.22	347	25.22	
21,001–24,000	2,160	26.16	1,797	26.12	363	26.38	
24,001–40,100	2,031	24.60	1,694	24.62	337	24.49	
≥40,001	1,983	24.02	1,654	24.04	329	23.91	
Urbanization^a^							0.989
High	5,525	66.92	4,602	66.89	923	67.08	
Medium	2,276	27.57	1,898	27.59	378	27.47	
Low	455	5.51	380	5.52	75	5.45	
CCI score^a, b^							0.993
0	4,404	53.34	3,670	53.34	734	53.34	
1	1,684	20.40	1,402	20.38	282	20.49	
≥2	2,168	26.26	1,808	26.28	360	26.16	
Comorbidities							
Diabetes mellitus							<0.001
No	7,529	91.19	6,225	90.48	1,304	94.77	
Yes	727	8.81	655	9.52	72	5.23	
Hypertension							0.126
No	7,418	89.85	6,166	89.62	1,252	90.99	
Yes	838	10.15	714	10.38	124	9.01	
Hyperlipidemia							<0.001
No	7,914	95.86	6,624	96.28	1,290	93.75	
Yes	342	4.14	256	3.72	86	6.25	
COPD^b^							0.003
No	7,921	95.94	6,581	95.65	1,340	97.38	
Yes	335	4.06	299	4.35	36	2.62	
Depression							<0.001
No	8,163	98.87	6,823	99.17	1,340	97.38	
Yes	93	1.13	57	0.83	36	2.62	
Anxiety							<0.001
No	7,879	95.43	6,622	96.25	1,257	91.35	
Yes	377	4.57	258	3.75	119	8.65	
Sleep disturbance							<0.001
No	7,636	92.49	6,433	93.50	1,203	87.43	
Yes	620	7.51	447	6.50	173	12.57	
Migraine							<0.001
No	8,204	99.37	6,852	99.59	1,352	98.26	
Yes	52	0.63	28	0.41	24	1.74	

^a^Matching variables.

^b^NMO, ICD-defined neuromyelitis optica; CCI, Charlson comorbidity index; COPD, chronic obstruction pulmonary disease.

Table 2 lists the adjusted HRs (aHRs) for CVD. After adjustment for relevant variables, NMO was associated with a significantly elevated risk of CVD (aHR = 1.40, 95% CI = 1.11–1.77). Risk factors significantly contributing to CVD included male (aHR = 1.33, 95% CI = 1.07–1.64), advanced age, and higher CCI scores. Furthermore, patients with comorbid diabetes (aHR = 1.41, 95% CI = 1.08–1.86), hyperlipidemia (aHR = 1.46, 95% CI = 1.04–2.04), COPD (aHR = 1.60, 95% CI = 1.12–2.27), or sleep disorders (aHR = 1.33, 95% CI = 1.01–1.74) demonstrated a higher risk to CVD events. Table 3 lists the aHRs for CBD. The association between NMO and CBD was particularly robust, with an aHR of 3.37 (95% CI = 2.69–4.22). Age was a critical determinant; individuals aged 45 and older faced a 4.71-fold higher risk of CBD. Significant comorbid contributors to CBD risk included diabetes (aHR = 1.46, 95% CI = 1.06–2.02), hypertension (aHR = 1.42, 95% CI = 1.07–1.89), hyperlipidemia (aHR = 1.72, 95% CI = 1.21–2.45), and anxiety (aHR = 1.65, 95% CI = 1.15–2.37).

**Table 2:** The incidence rate and risk of incident cardiovascular disease.

Variables	Cardiovascular disease			
	No. of events (%)	IR^a^	aHR (95% CI)^a^	*p*-value
Total	440 (5.33)	7.69		
Patient cohort				
Comparison	347 (5.04)	7.24	Reference	
NMO ^a^	93 (6.76)	10.02	1.40 (1.11–1.77)	0.005
Sex				
Female	313 (5.00)	7.19	Reference	
Male	127 (6.37)	9.28	1.33 (1.07–1.64)	0.009
Age (year)				
<25	30 (1.98)	2.71	Reference	
25–34	64 (3.82)	5.12	1.89 (1.22–2.94)	0.005
35–44	127 (5.83)	7.93	2.66 (1.78–3.98)	<0.001
≥45	219 (7.58)	12.46	3.58 (2.41–5.34)	<0.001
Insured salary (NTD)				
≤21,000	145 (6.96)	8.21	Reference	
21,001–24,000	106 (4.91)	8.30	0.93 (0.72–1.20)	0.562
24,001–40,100	80 (3.94)	5.79	0.71 (0.54–0.94)	0.017
≥40,001	109 (5.50)	8.43	0.97 (0.75–1.25)	0.787
Urbanization				
High	297 (5.38)	7.84	Reference	
Medium	121 (5.32)	7.91	0.95 (0.70–1.28)	0.602
Low	22 (4.84)	7.27	0.71 (0.33–1.61)	0.451
CCI score^a^				
0	156 (3.54)	4.95	Reference	
1	98 (5.82)	8.14	1.26 (0.97–1.64)	0.087
≥2	186 (8.58)	13.67	1.88 (1.48–2.40)	<0.001
Comorbidities				
Diabetes mellitus	76 (10.45)	17.63	1.41 (1.08–1.86)	0.013
Hypertension	80 (9.55)	15.83	1.26 (0.97–1.64)	0.085
Hyperlipidemia	40 (11.70)	18.20	1.46 (1.04–2.04)	0.029
COPD ^a^	36 (10.75)	16.62	1.60 (1.12–2.27)	0.009
Depression	8 (8.60)	12.18	1.04 (0.51–2.12)	0.918
Anxiety	30 (7.96)	10.89	1.05 (0.71–1.53)	0.821
Sleep disturbance	70 (11.29)	12.41	1.33 (1.01–1.74)	0.039
Migraine	5 (9.62)	12.59	1.72 (0.70–4.19)	0.234

^a^IR, incident rate per 1,000 person-year; aHR, adjusted hazard ratio; NMO, ICD-defined neuromyelitis optica; CCI, Charlson comorbidity index; COPD, chronic obstruction pulmonary disease.

**Table 3:** The incidence rate and risk of incident cerebrovascular disease.

Variables	Cerebrovascular disease			
	No. of events (%)	IR^a^	aHR (95% CI)^a^	*p*-value
Total	332 (4.02)	5.73		
Patient cohort				
Comparison	200 (2.91)	4.10	Reference	
NMO ^a^	132 (9.59)	14.44	3.37 (2.69–4.22)	<0.001
Sex				
Female	225 (3.59)	5.10	Reference	
Male	107 (5.36)	7.74	1.59 (1.26–2.02)	<0.001
Age (year)				
<25	19 (1.25)	1.71	Reference	
25–34	35 (2.09)	2.78	1.58 (0.90–2.78)	0.113
35–44	94 (4.32)	5.78	3.11 (1.89–5.11)	<0.001
≥45	184 (6.37)	10.24	4.71 (2.88–7.68)	<0.001
Insured salary (NTD)				
≤21,000	107 (5.14)	5.96	Reference	
21,001-24,000	75 (3.47)	5.79	0.87 (0.65–1.18)	0.385
24,001–40,100	78 (3.84)	5.63	0.97 (0.72–1.30)	0.824
≥40,001	72 (3.63)	5.48	0.84 (0.62–1.14)	0.270
Urbanization				
High	213 (3.86)	5.53	Reference	
Medium	103 (4.53)	6.58	1.23 (0.87–1.74)	0.234
Low	16 (3.52)	4.27	0.82 (0.35–2.23)	0.610
CCI score^a^				
0	128 (2.91)	4.03	Reference	
1	77 (4.57)	6.29	1.22 (0.91–1.64)	0.183
≥2	127 (5.86)	9.13	1.49 (1.13–1.97)	0.005
Comorbidities				
Diabetes mellitus	54 (7.43)	12.25	1.46 (1.06–2.02)	0.021
Hypertension	69 (8.23)	13.14	1.42 (1.07–1.89)	0.016
Hyperlipidemia	39 (11.4)	17.46	1.72 (1.21–2.45)	0.002
COPD^a^	12 (3.58)	5.21	0.66 (0.37–1.19)	0.166
Depression	8 (8.60)	12.17	1.23 (0.60–2.52)	0.568
Anxiety	36 (9.55)	12.92	1.65 (1.15–2.37)	0.006
Sleep disturbance	58 (9.35)	9.99	1.31 (0.97–1.76)	0.079
Migraine	5 (9.62)	12.67	2.03 (0.83–4.97)	0.121

^a^IR, incident rate per 1,000 person-year; aHR, adjusted hazard ratio; NMO, ICD-defined neuromyelitis optica; CCI, Charlson comorbidity index; COPD, chronic obstruction pulmonary disease.

Table 4 illustrates the associations of NMO severity with CVD and CBD risk. Analysis by disease severity revealed that NMO patients with the highest hospitalization ratios faced the greatest risks for both CVD (aHR = 2.23, 95% CI = 1.27–3.17) and CBD (aHR = 7.01, 95% CI = 5.26–9.32). While CBD risk remained significant even among patients with mild or no hospitalization, the elevated risk for CVD was primarily confined to those with more severe disease. Table 5 presents the HRs for specific CVD and CBD conditions. Regarding specific events, NMO was significantly linked to ischemic stroke (aHR = 3.32, 95% CI = 2.63–4.20), hemorrhagic stroke (aHR = 3.90, 95% CI = 2.74–5.55), and TIA (aHR = 2.21, 95% CI = 1.02–4.80). No significant correlation was observed between NMOSD and acute myocardial infarction, atrial fibrillation, coronary artery disease, or heart failure.

**Table 4:** Subgroup analysis by the severity of ICD-defined neuromyelitis optica.

Variables	Cardiovascular disease		Cerebrovascular disease	
	aHR (95% CI)^a^	*p*-value	aHR (95% CI) ^a^	*p*-value
By the total length of hospital stay				
Mild	1.21 (0.87–1.69)	0.262	2.48 (1.80–3.42)	<0.001
Moderate	0.98 (0.60–1.60)	0.933	1.63 (0.99–2.68)	0.057
Severe	2.23 (1.27–3.17)	<0.001	7.01 (5.26–9.32)	<0.001
By stratification with hospitalization				
Without hospitalization	1.41 (0.92–2.16)	0.119	3.23 (2.20–4.73)	<0.001
With hospitalization	1.40 (1.08–1.82)	0.012	3.41 (2.67–4.36)	<0.001

^a^aHR, adjusted hazard ratio. Adjustment for sex, age, insured salary, urbanization, CCI score, and all comorbidities.

**Table 5:** Risk of incident each cardiovascular and cerebrovascular disease.

Variables	Comparison		NMO^a^		NMO vs. Comparison (ref.)	
	No. of events (%)	IR^a^	No. of events (%)	IR^a^	aHR (95% CI)^b^	*p*-value
Cardiovascular disease						
AMI ^a^	26 (0.38)	0.53	5 (0.36)	0.51	1.21 (0.46–3.19)	0.707
Atrial fibrillation	34 (0.49)	0.69	5 (0.36)	0.51	0.75 (0.29–1.95)	0.560
Coronary artery disease	294 (4.27)	6.11	68 (4.94)	7.25	1.18 (0.90–1.54)	0.230
Heart failure	91 (1.32)	1.85	23 (1.67)	2.38	1.48 (0.93–2.36)	0.102
Cerebrovascular disease						
Ischemic stroke	184 (2.67)	3.77	122 (8.87)	13.24	3.32 (2.63–4.20)	<0.001
Hemorrhagic stroke	74 (1.08)	1.50	58 (4.22)	6.10	3.90 (2.74–5.55)	<0.001
TIA ^a^	21 (0.31)	0.42	10 (0.73)	1.03	2.21 (1.02–4.80)	0.045

^a^NMO, ICD-defined neuromyelitis optica; IR, incident rate per 1,000 person-year; AMI, acute myocardial infarction; TIA, transient ischemic attack.

^b^aHR, adjusted hazard ratio. Adjustment for sex, age, insured salary, urbanization, CCI score, and all comorbidities.

## 4 Discussion

In this large population-based cohort study, patients with ICD-defined NMO had a clearly elevated risk of CBD, including ischemic stroke, hemorrhagic stroke, and TIA, among which hemorrhagic stroke posed the greatest risk. However, NMO did not increase the likelihood of CVD, including myocardial infarction, atrial fibrillation, coronary artery disease, and heart failure.

NMO has been reported worldwide and is associated with poor prognosis. NMO relapses typically worsen progressively over several days before reaching the peak clinical deficit and then gradually improving over the subsequent weeks or months. However, recovery is often incomplete, and many patients experience early cumulative disability due to frequent and severe relapses (20).

Although astrocytes maintain BBB integrity, polymorphonuclear leukocytes—not astrocyte loss—primarily drive BBB disruption. Consequently, leukocyte depletion restores BBB integrity, preventing astrocyte damage and permitting their repopulation (21). A recent study on neuromyelitis optica spectrum disorder (NMOSD) indicated that increased BBB permeability allows circulating AQP4 autoantibodies to infiltrate the central nervous system. The study also highlighted the role of glucose-regulated protein autoantibodies as BBB-reactive agents that contribute to antibody-induced BBB dysfunction (12).

The risk of CBD in patients with NMO remains insufficiently understood and investigated. Therefore, we examined whether CBD risk varied between individuals with ICD-defined NMO and a matched population without NMO in Taiwan. We observed an increased risk of various CBDs in patients with NMO, including ischemic stroke (aHR = 3.32), hemorrhagic stroke (aHR = 3.90), and TIA (aHR = 2.21), among which the highest risk was noted for hemorrhagic stroke. Previous pathological studies have demonstrated thickened and hyalinized small blood vessels and perivascular inflammation characterized by the deposition of IgG and complement within demyelinating lesions in NMOSD (22). Pathologies affecting small vessels can cause both ischemic and hemorrhagic outcomes. Moreover, inflammatory vascular markers associated with endothelial dysfunction are elevated in NMOSD during acute relapses, similar to observations reported in MS (23, 24). A case of NMOSD with recurrent intracranial hemorrhage was previously reported, suggesting a potential link between NMOSD and cerebellar vascular dysfunction (25). Several cases of acute ischemic or hemorrhagic stroke have also been reported during intravenous high-dose steroid treatment (14, 15). In a Korean cohort study, stroke risk was higher in patients with NMOSD than in matched controls (16).

From a mechanistic perspective, NMOSD is characterized by systemic inflammation and immune-mediated astrocytopathy, in which pathogenic AQP4-IgG binds to astrocytic AQP4 and promotes complement-dependent astrocytic injury, a central pathological feature of AQP4-IgG–positive NMOSD (26, 27). These inflammatory pathways, together with complement activation, may contribute to vascular pathology by promoting endothelial dysfunction, vascular inflammation, and subsequent vascular injury (28). In addition, proinflammatory cytokines and activated immune cells, including neutrophils, may promote a prothrombotic state through multiple mechanisms, including upregulation of tissue factor expression, impairment of endogenous anticoagulant pathways, and dysregulation of fibrinolysis (29). Neutrophil activation and the formation of neutrophil extracellular traps (NETs) may contribute to thrombosis by promoting platelet aggregation and activation of the coagulation cascade (30), thereby amplifying thromboinflammatory processes and facilitating thrombogenesis in NMOSD. These processes may collectively increase susceptibility to thrombotic events. Consistent with this biological plausibility, a nationwide cohort study from South Korea reported an increased risk of stroke among patients with NMOSD (16).

The relationship between CVD risk and NMO remains poorly understood and underexplored. To address this gap, we examined the association between NMO and CVD risk using data from Taiwan’s NHIRD. Patients with ICD-defined NMO in our cohort did not have a higher risk of myocardial infarction, atrial fibrillation, *coronary artery disease*, or heart failure. By contrast, in a previous cohort study conducted in Korea, the risk of myocardial infarction was elevated in both MS and NMOSD and remained similar between both conditions (17). Thus, the association between NMO and CVD risk remains unclear, highlighting the need for further investigation in large-scale studies.

In our sample, patients with certain comorbidities, including diabetes mellitus, hypertension, hyperlipidemia, and anxiety disorder, exhibited an increased risk of stroke. Additionally, stroke risk was higher among male and older patients. A study analyzing data from a U. S. insurance claims database revealed that several comorbidities, including hypertension and diabetes mellitus, were more common in patients with NMO than those without NMO (31). In another study of patients with NMO, elevated serum levels of low-density lipoprotein cholesterol were independently and positively associated with disease relapse (32). Patients exhibited a higher risk of developing diabetes mellitus than those with MS. However, this increased risk was primarily associated with prolonged or frequent steroid use, rendering the directness of the connection to NMO pathogenesis unclear. Severe disability in NMO has also been correlated with an elevated risk of diabetes mellitus (33). Previous studies indicated that autonomic dysfunction is commonly observed in both MS and NMOSD, involving cardiovascular, thermoregulation, and fatigue-related symptoms (34, 35). Moreover, NMOSD-related disability and decreased mobility may exacerbate risk factors and promote a sedentary lifestyle, thereby increasing stroke risk. This phenomenon may explain why a higher incidence of stroke and stroke-related complications has sometimes been observed in patients with NMOSD. Additionally, depression, anxiety, and sleep disturbances are highly prevalent among individuals with NMOSD (36, 37).

In our sample, CBD risk was significantly associated with ICD-defined NMO severity, and the highest risk was observed in patients with severe NMO. CBD risk was elevated in both hospitalized and nonhospitalized patients with NMO, although hospitalized patients exhibited a higher risk. A previous NMOSD study pointed out that most NMOSD patients experience frequent relapses that cause more severe, longer-lasting deficits than MS. Managing these flare-ups typically requires hospitalization and immunomodulatory therapy. However, readmission rates peak shortly after discharge, with half occurring within 13 days—primarily driven by neurological complications (50.2%) (38, 39).

Our findings revealed a higher risk of various CBDs in patients with ICD-defined NMO. However, patients with ICD-defined NMO in our sample did not have an increased risk of CVDs, such as myocardial infarction, atrial fibrillation, coronary artery disease, or heart failure. Studies have suggested that AQP4 antibodies play a key role in NMOSD-related BBB dysfunction (12, 40, 41). In turn, antibody-mediated BBB impairment in NMOSD (14) may make patients with NMOSD more susceptible to CBD than CVD. The present study found that patients with ICD-defined NMO have an increased risk of CBD, including ischemic stroke, TIA, and hemorrhagic stroke, which presents the highest risk. However, ICD-defined NMO does not appear to elevate CVD risk, including the risk of myocardial infarction, atrial fibrillation, coronary artery disease, and heart failure. Our findings highlight the need for medical professionals to recognize the possibility of acute macrovascular events when patients with ICD-defined NMO experience the sudden onset or worsening of new or existing symptoms.

However, these mechanistic interpretations should be approached with caution. The present study was based on administrative claims data from the NHIRD, which does not include biomarker, imaging, or laboratory information (e.g., inflammatory markers, coagulation profiles, or neuroimaging findings) necessary to directly evaluate these pathways. Therefore, the proposed mechanisms remain hypothetical and should be considered hypothesis-generating rather than confirmatory. Importantly, without serological data regarding AQP4-IgG and MOG-IgG status, we could not distinguish between contemporary definitions of NMOSD and MOGAD, nor could we further categorize patients by their specific clinical phenotypes (such as the presence of optic neuritis, longitudinal extensive transverse myelitis, or specific cerebral lesions). Given that NMO and MOGAD are now recognized as distinct immunological entities with different target cells, our reliance on historical, ICD-defined cohorts inherently introduces population heterogeneity. Therefore, the proposed macrovascular mechanisms remain hypothetical and should be considered hypothesis-generating rather than confirmatory.

The lack of smoking and BMI data might lead to an overestimation of the risk (positive bias), as these factors are prevalent in patients with chronic inflammatory conditions. To address the potential detection bias, we utilized relevant clinical diagnoses as surrogate variables. For example, COPD was used as an indicator of heavy smoking, while hypertension, dyslipidemia, and diabetes were included to address metabolic risks. As highlighted in Tables 2, 3, these comorbidities emerged as significant predictors of CVD and CBD, demonstrating that our model effectively accounted for the influence of established risk factors. Furthermore, adjustments were made for the CCI score and key comorbidities, which reflect both the patients’ overall health status and their interaction frequency with the healthcare system.

This population-based cohort study has several notable strengths. First, we employed a nationwide, population-based design, selecting patients from the entire Taiwanese population and monitoring them over an extended follow-up period. This methodology provided a large, representative sample with substantial statistical reliability and reduced the selection bias prevalent in observational research. Additionally, the large sample size allowed for subgroup stratification during statistical analysis, enabling us to thoroughly evaluate the effects of NMOSD on the risk of macrovascular events. Second, in Taiwan, insurance claims for in-hospital treatments are stringently monitored and audited under the NHI system. This rigorous surveillance program improves the reliability of diagnoses derived from insurance claims. Third, all patients with NMOSD and age- and gender-matched controls were selected from a nationally representative dataset.

This study also has several limitations. Therefore, certain confounding variables may not have been properly controlled for in this study. First, we were unable to collect data on factors associated to the risk of macrovascular events, including smoking status, alcohol use, body mass index, physical activity, personal medical history, disease activity, and disease duration. Second, a major limitation inherent to the claims-based nature of the NHIRD is the lack of detailed laboratory, serological, and neuroimaging data. Consequently, we could not classify patients by their immunological subtypes (e.g., AQP4-IgG vs. MOG-IgG status) or further categorize them by clinical phenotypes, such as the presence or absence of optic neuritis, longitudinally extensive transverse myelitis (LETM), or specific cerebral lesions. Crucially, our study period spanned from 2003 to 2020, capturing patients based on ICD-9-CM and ICD-10-CM administrative codes before the formulation of the 2023 international diagnostic criteria for MOGAD. Therefore, our cohort reflects an administrative and historical definition of NMO/NMOSD, which likely contains individuals who would currently be diagnosed with MOGAD rather than NMOSD. Nevertheless, prior epidemiological studies in Asian populations indicate that AQP4-IgG seropositivity accounts for the vast majority (approximately 70–80%) of cases historically coded as NMO, whereas MOGAD represents a small fraction (42). Although this heterogeneity is likely non-differential for macrovascular outcomes, whether distinct clinical presentations carry varying risks remains unclear. Future prospective cohorts incorporating precise clinic-immunological, imaging, and molecular classifications are warranted to confirm these associations.

## Data Availability

The data analyzed in this study is subject to the following licenses/restrictions: the data that support the findings of this study are available from HWDC, MOHW but restrictions apply to the availability of these data, which were used under license for the current study, and so are not publicly available. Requests to access these datasets should be directed to HWDC, MOHW (https://dep.mohw.gov.tw/dos/np-2497-113.html).

## References

[1] Carnero Contentti E Correale J. Neuromyelitis optica spectrum disorders: from pathophysiology to therapeutic strategies. J Neuroinflammation. (2021) 18:208. doi: 10.1186/s12974-021-02249-1, 34530847 PMC8444436

[2] Wingerchuk DM Lennon VA Lucchinetti CF Pittock SJ Weinshenker BG. The spectrum of neuromyelitis optica. Lancet Neurol. (2007) 6:805–15. doi: 10.1016/S1474-4422(07)70216-8, 17706564

[3] Ciccarelli O Cohen JA Reingold SC Weinshenker BG International Conference on SpinalCord I, Imaging in MultipleS . Spinal cord involvement in multiple sclerosis and neuromyelitis optica spectrum disorders. Lancet Neurol. (2019) 18:185–97. doi: 10.1016/S1474-4422(18)30460-5 30663608

[4] Lennon VA Wingerchuk DM Kryzer TJ Pittock SJ Lucchinetti CF Fujihara K . A serum autoantibody marker of neuromyelitis optica: distinction from multiple sclerosis. Lancet. (2004) 364:2106–12. doi: 10.1016/S0140-6736(04)17551-X, 15589308

[5] Lennon VA Kryzer TJ Pittock SJ Verkman AS Hinson SR. IgG marker of optic-spinal multiple sclerosis binds to the aquaporin-4 water channel. J Exp Med. (2005) 202:473–7. doi: 10.1084/jem.20050304, 16087714 PMC2212860

[6] Kitley J Waters P Woodhall M Leite MI Murchison A George J . Neuromyelitis optica spectrum disorders with aquaporin-4 and myelin-oligodendrocyte glycoprotein antibodies: a comparative study. JAMA Neurol. (2014) 71:276–83. doi: 10.1001/jamaneurol.2013.5857, 24425068

[7] Jarius S Paul F Aktas O Asgari N Dale RC de Seze J . MOG encephalomyelitis: international recommendations on diagnosis and antibody testing. J Neuroinflammation. (2018) 15:134. doi: 10.1186/s12974-018-1144-2, 29724224 PMC5932838

[8] Banwell B Bennett JL Marignier R Kim HJ Brilot F Flanagan EP . Diagnosis of myelin oligodendrocyte glycoprotein antibody-associated disease: international MOGAD panel proposed criteria. Lancet Neurol. (2023) 22:268–82. doi: 10.1016/S1474-4422(22)00431-8, 36706773

[9] Kumpfel T Giglhuber K Aktas O Ayzenberg I Bellmann-Strobl J Haussler V . Update on the diagnosis and treatment of neuromyelitis optica spectrum disorders (NMOSD) - revised recommendations of the Neuromyelitis Optica study group (NEMOS). Part II: attack therapy and long-term management. J Neurol. (2024) 271:141–76. doi: 10.1007/s00415-023-11910-z, 37676297 PMC10770020

[10] Zekeridou A Lennon VA. Aquaporin-4 autoimmunity. Neurol Neuroimmunol Neuroinflamm. (2015) 2:e110. doi: 10.1212/NXI.0000000000000110, 26185772 PMC4442096

[11] Corbali O Chitnis T. Pathophysiology of myelin oligodendrocyte glycoprotein antibody disease. Front Neurol. (2023) 14:1137998. doi: 10.3389/fneur.2023.1137998, 36925938 PMC10011114

[12] Shimizu F Nakamori M. Blood-brain barrier disruption in Neuroimmunological disease. Int J Mol Sci. (2024) 25:10625. doi: 10.3390/ijms251910625, 39408955 PMC11476930

[13] Geraldes R Esiri MM Perera R Yee SA Jenkins D Palace J . Vascular disease and multiple sclerosis: a post-mortem study exploring their relationships. Brain. (2020) 143:2998–3012. doi: 10.1093/brain/awaa255, 32875311

[14] Wang ZY Wang M Guo JJ Gao YL Yu XF. Acute bilateral cerebral infarction in the presence of neuromyelitis optica spectrum disorder: a case report. Medicine (Baltimore). (2020) 99:e22616. doi: 10.1097/MD.0000000000022616, 33019482 PMC7535662

[15] Kamo H Ueno Y Sugiyama M Miyamoto N Yamashiro K Tanaka R . Pontine hemorrhage accompanied by neuromyelitis optica spectrum disorder. J Neuroimmunol. (2019) 330:19–22. doi: 10.1016/j.jneuroim.2019.01.020, 30769213

[16] Cho EB Yeo Y Jung JH Jeong SM Han KD Shin DW . Risk of stroke in multiple sclerosis and neuromyelitis optic spectrum disorder: a Nationwide cohort study in South Korea. J Neurol Neurosurg Psychiatry. (2022) 93:1146–53. doi: 10.1136/jnnp-2022-329628, 36028308

[17] Cho EB Yeo Y Jung JH Jeong SM Han K Yang JH . Acute myocardial infarction risk in multiple sclerosis and neuromyelitis optica spectrum disorder: a nationwide cohort study in South Korea. Mult Scler. (2022) 28:1849–58. doi: 10.1177/13524585221096964, 35695204

[18] Hsieh CY Su CC Shao SC Sung SF Lin SJ Kao Yang YH . Taiwan's National Health Insurance Research Database: past and future. Clin Epidemiol. (2019) 11:349–58. doi: 10.2147/CLEP.S196293, 31118821 PMC6509937

[19] Lai SW Liao KF Lin CL Lin CC Lin CH. Longitudinal data of multimorbidity and polypharmacy in older adults in Taiwan from 2000 to 2013. Biomedicine (Taipei). (2020) 10:1–4. doi: 10.37796/2211-8039.1013, 33854914 PMC7608846

[20] Wingerchuk DM Hogancamp WF O'Brien PC Weinshenker BG. The clinical course of neuromyelitis optica (Devic's syndrome). Neurology. (1999) 53:1107–14. doi: 10.1212/WNL.53.5.1107, 10496275

[21] Winkler A Wrzos C Haberl M Weil MT Gao M Mobius W . Blood-brain barrier resealing in neuromyelitis optica occurs independently of astrocyte regeneration. J Clin Invest. (2021) 131:e141694. doi: 10.1172/JCI141694, 33645550 PMC7919716

[22] Lucchinetti CF Mandler RN McGahern D Bruck W Gleich G Ransohoff RM . A role for humoral mechanisms in the pathogenesis of Devic's neuromyelitis optica. Brain. (2002) 125:1450–61. doi: 10.1093/brain/awf151 12076996 PMC5444467

[23] Yi M Liu MQ Chou LS Jiang SM Zhang LJ Huang CN . Correlation between serum levels of endothelin-1 and disease severity in patients with neuromyelitis optica spectrum disorders. Immunobiology. (2020) 225:151959. doi: 10.1016/j.imbio.2020.151959, 32517881

[24] Wang H Wang K Wang C Zhong X Qiu W Hu X. Increased plasma levels of pentraxin 3 in patients with multiple sclerosis and neuromyelitis optica. Mult Scler. (2013) 19:926–31. doi: 10.1177/1352458512457845, 23012252

[25] Yaguchi H Mito Y Ohashi I Nomura T Yabe I Tajima Y. Neuromyelitis Optica Spectrum disorder with recurrent intracranial hemorrhage. Intern Med. (2017) 56:1729–32. doi: 10.2169/internalmedicine.56.7889, 28674367 PMC5519480

[26] Asavapanumas N Tradtrantip L Verkman AS. Targeting the complement system in neuromyelitis optica spectrum disorder. Expert Opin Biol Ther. (2021) 21:1073–86. doi: 10.1080/14712598.2021.1884223, 33513036 PMC8316261

[27] Takai Y Misu T Suzuki H Takahashi T Okada H Tanaka S . Staging of astrocytopathy and complement activation in neuromyelitis optica spectrum disorders. Brain. (2021) 144:2401–15. doi: 10.1093/brain/awab102, 33711152

[28] Davis C Fischer J Ley K Sarembock IJ. The role of inflammation in vascular injury and repair. J Thromb Haemost. (2003) 1:1699–709. doi: 10.1046/j.1538-7836.2003.00292.x, 12911580

[29] Furie B Furie BC. Mechanisms of thrombus formation. N Engl J Med. (2008) 359:938–49. doi: 10.1056/NEJMra0801082, 18753650

[30] Zhou Y Xu Z Liu Z. Impact of neutrophil extracellular traps on thrombosis formation: new findings and future perspective. Front Cell Infect Microbiol. (2022) 12:910908. doi: 10.3389/fcimb.2022.910908, 35711663 PMC9195303

[31] Ajmera MR Boscoe A Mauskopf J Candrilli SD Levy M. Evaluation of comorbidities and health care resource use among patients with highly active neuromyelitis optica. J Neurol Sci. (2018) 384:96–103. doi: 10.1016/j.jns.2017.11.022, 29249387

[32] Ding J Chen FP Song YY Li HY Ai XW Chen Y . Serum low-density lipoprotein cholesterol levels are associated with relapse in Neuromyelitis Optica Spectrum disorder. J Inflamm Res. (2024) 17:8227–40. doi: 10.2147/JIR.S489723, 39525310 PMC11549894

[33] Cho EB Han K Jung JH Chung YH Kwon S Park J . The risk of type 2 diabetes mellitus in multiple sclerosis and neuromyelitis optica spectrum disorder: a nationwide cohort study. Mult Scler Relat Disord. (2024) 85:105519. doi: 10.1016/j.msard.2024.105519, 38457883

[34] Crnosija L Krbot Skoric M Andabaka M Junakovic A Martinovic V Ivanovic J . Autonomic dysfunction in people with neuromyelitis optica spectrum disorders. Mult Scler. (2020) 26:688–95. doi: 10.1177/1352458519837703, 30887872

[35] Valdivia-Tangarife ER Gamez-Nava JI Cortes-Enriquez F Mireles-Ramirez MA Gonzalez-Lopez L Saldana-Cruz AM . Risk factors associated with permanent disability in neuromyelitis optica spectrum disorders. Mult Scler Relat Disord. (2022) 68:104114. doi: 10.1016/j.msard.2022.104114, 36037753

[36] Deng J Zhou F Hou W Silver Z Wong CY Chang O . The prevalence of depression, anxiety, and sleep disturbances in COVID-19 patients: a meta-analysis. Ann N Y Acad Sci. (2021) 1486:90–111. doi: 10.1111/nyas.14506, 33009668 PMC7675607

[37] Dong JY Zhang YH Tong J Qin LQ. Depression and risk of stroke: a meta-analysis of prospective studies. Stroke. (2012) 43:32–7. doi: 10.1161/STROKEAHA.111.630871, 22020036

[38] Houzen H Kondo K Niino M Horiuchi K Takahashi T Nakashima I . Prevalence and clinical features of neuromyelitis optica spectrum disorders in northern Japan. Neurology. (2017) 89:1995–2001. doi: 10.1212/WNL.0000000000004611, 28986408

[39] Padarti A Amritphale A Kilgo W. Hospital readmission rates in patients with Neuromyelitis Optica Spectrum disorder. Int J MS Care. (2023) 25:221–5. doi: 10.7224/1537-2073.2022-049, 37720258 PMC10503814

[40] Zhu W Zhang Y Wang Z Fu Y Yan Y. Monoclonal antibody-based treatments for Neuromyelitis Optica Spectrum disorders: from bench to bedside. Neurosci Bull. (2020) 36:1213–24. doi: 10.1007/s12264-020-00525-3, 32533450 PMC7532256

[41] Vincent T Saikali P Cayrol R Roth AD Bar-Or A Prat A . Functional consequences of neuromyelitis optica-IgG astrocyte interactions on blood-brain barrier permeability and granulocyte recruitment. J Immunol. (2008) 181:5730–7. doi: 10.4049/jimmunol.181.8.5730, 18832732

[42] Hor JY Wong CK Ew JV Idris S Tan HJ Wong DYJ. Neuromyelitis optica spectrum disorder in Asia: epidemiology and risk factors. Neurol Clin Neurosci. (2021) 9:274–81. doi: 10.1111/ncn3.12478

